# Supplementary material for: A melanopsin ganglion cell subtype forms a dorsal retinal mosaic projecting to the supraoptic nucleus
Source: Nat Commun. 2023 Mar 17;14:1492. doi: 10.1038/s41467-023-36955-6 (PMC10023714; doi:10.1038/s41467-023-36955-6)
Supplement: Supplementary file 1 — Supplementary Information [file 41467_2023_36955_MOESM1_ESM.pdf]

A melanopsin ganglion cell subtype forms a dorsal retinal mosaic projecting to the supraoptic nucleus

Supplementary Information

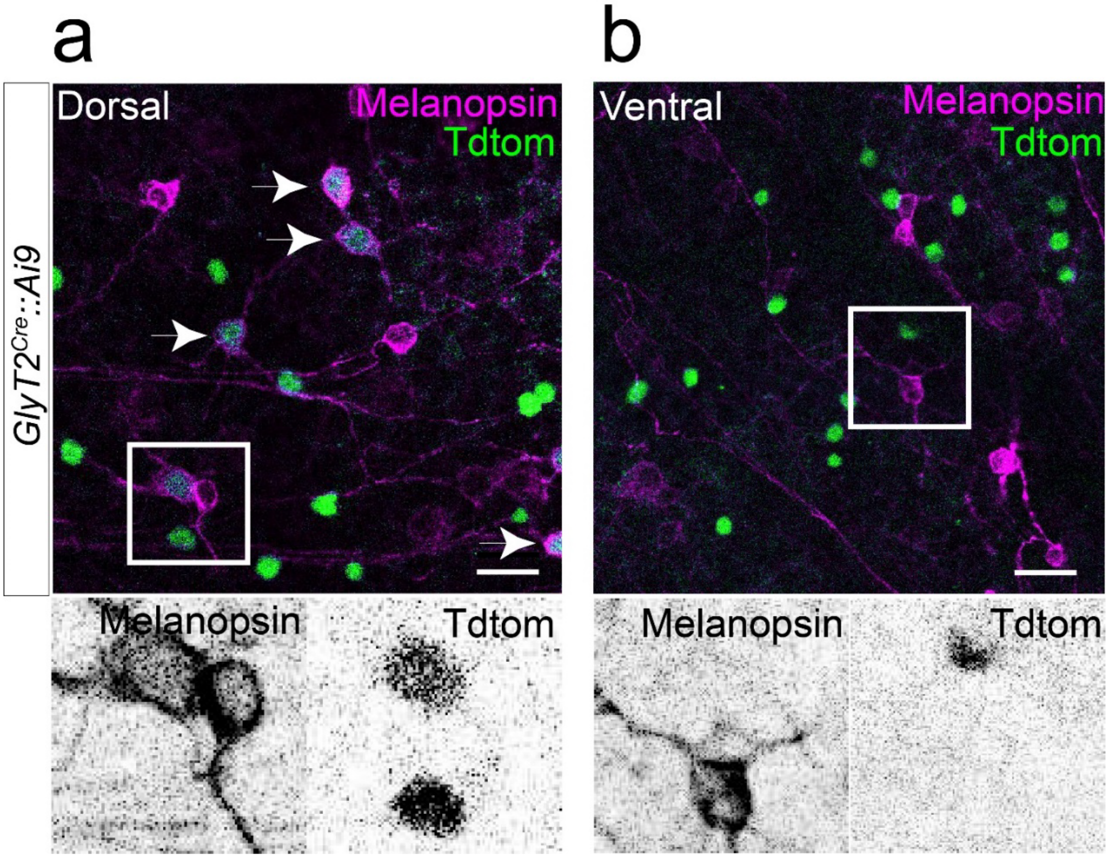

Supplementary Figure 1. Fluorescent IpRGCs in the dorsal retina of the *GlyT2^Cre^;Ai9* mice.

Confocal images of melanopsin expressing Tdtomato-positive ipRGCs in the dorsal **(a)** but not the ventral **(b)** hemisphere of the wholemount retina of the *GlyT2*^{Cre}*;Ai9* mouse. Scale bar in a,b = 25 µm.

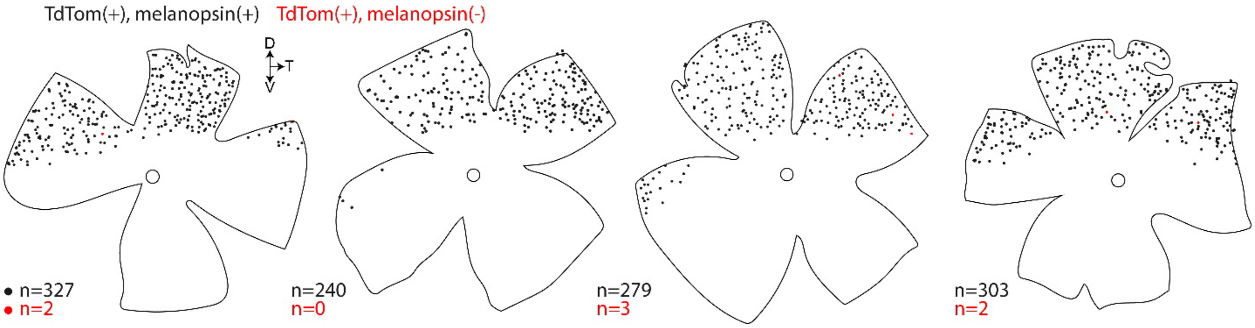

Supplementary Figure 2. Retina maps of RGCs labeled in the *GlyT2^{Cre};Ai9* retina. Distribution maps of RBPMS-positive, TdTomato-positive cells labeled the *GlyT2^{Cre}* mouse. Each cell assessed for the presence or absence of melanopsin co-staining. Source data are provided as a source data file.

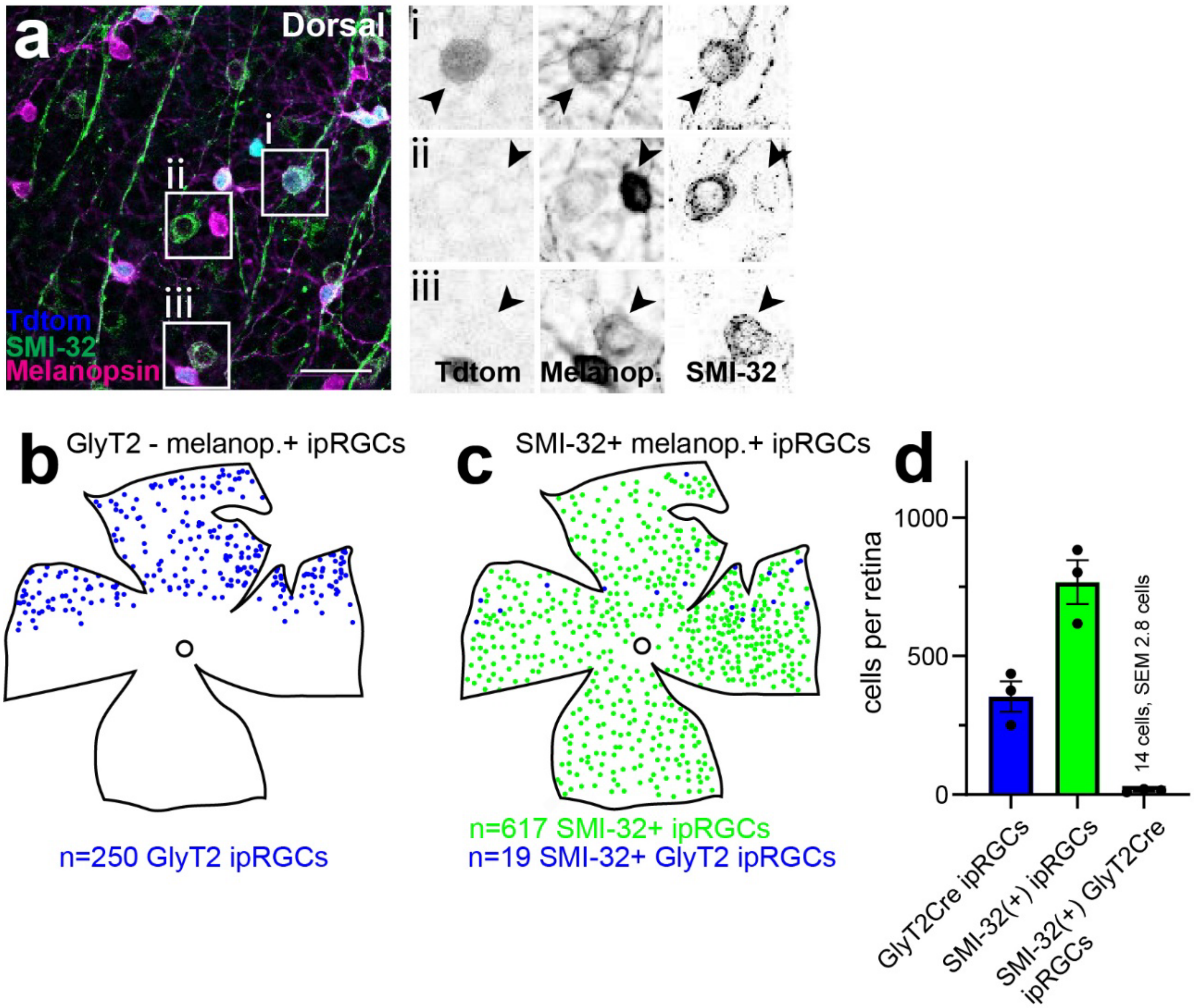

Supplementary Figure 3. Distribution and quantification of SMI-32-positive ipRGCs in the *GlyT2^{Cre};Ai9* retina.

(a) Confocal images of *GlyT2^{Cre};Ai9* retina immunostained for SMI-32 and melanopsin. Scale bar =25 µm. **(b)** Representative retina distribution map of *GlyT2^{Cre}* ipRGCs (TdTomato-positive & melanopsin-positive compared with the **(c)** distribution map of M4 ipRGCs (Melanopsin-positive & SMI-32-positive) within the same retina. Blue dots identify the distribution of putative *GlyT2^{Cre}*-positive M4 ipRGCs (SMI-32-positive TdTomato-positive, melanopsin-positive). **(d)** quantification of *GlyT2^{Cre}* ipRGCs, M4/SMI-32-positive ipRGCs, and M4/SMI-32 ipRGCs labeled in the *GlyT2^{Cre}* mouse line (n=3 retinas from n=3 mice; data are presented as mean values ± SEM). Source data are provided as a source data file.

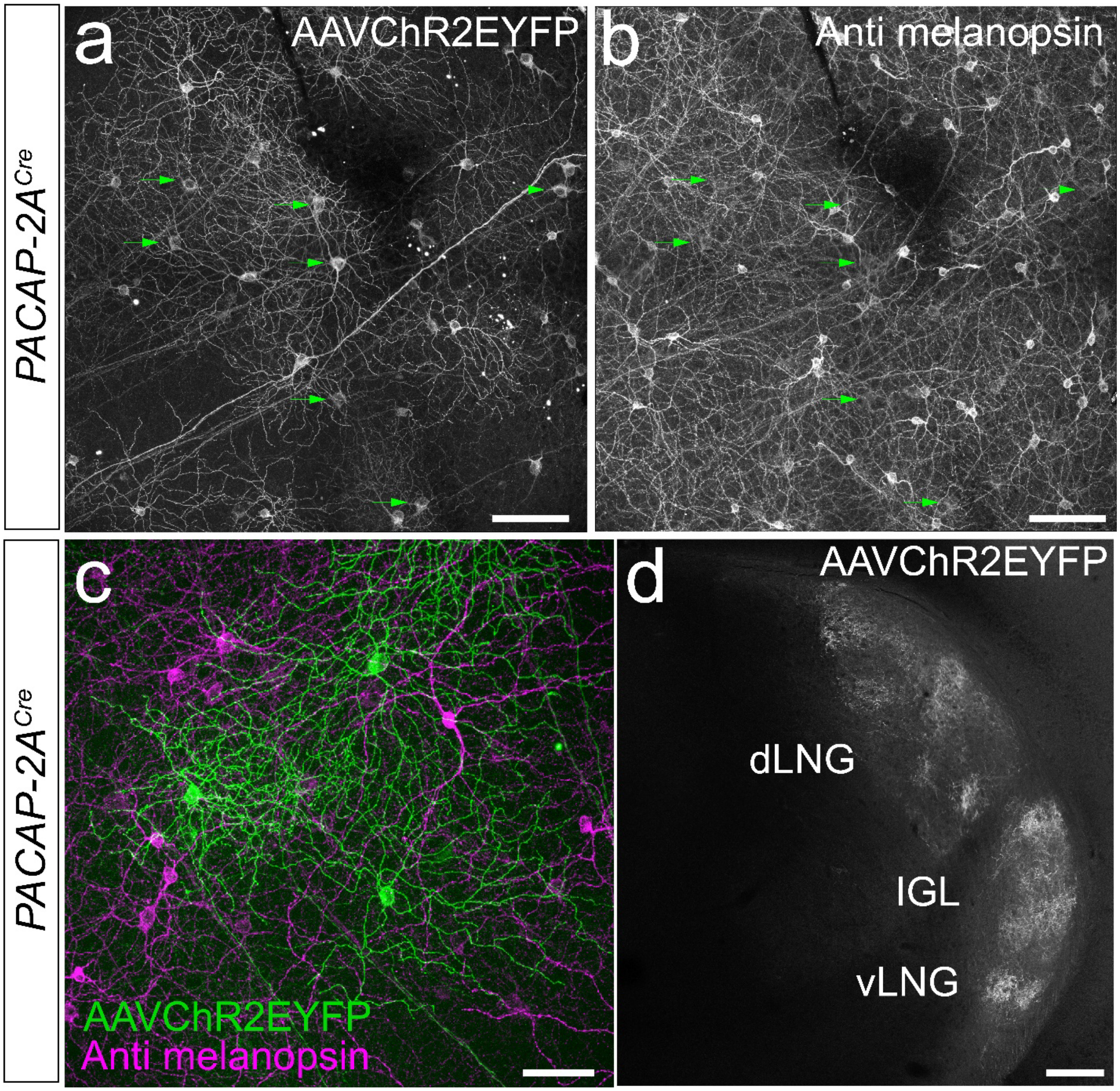

Supplementary Figure 4. PACAP-2A Cre RGCs in the mouse retina.
We made a Cre-dependent AAV-ChR2^{EYFP} injection into the eye of a *PACAP-2A*^{Cre} mouse which resulted in labelling of a diverse array of RGCs. **(a-b)** Most RGCs labeled in this line were melanopsin negative, aside from some weakly melanopsin positive ipRGCs with large somas which we presume are M4 ipRGCs (green arrows). **(c)** The morphology of most RGCs labeled in this line resembled conventional RGCs and there was no EYFP-positive and strongly melanopsin-immunopositive ipRGCs. **(d)** Central projections of PACAP-positive RGCs are primarily to image-forming regions of the LGN and avoid the IGL suggesting that in the mouse retina PACAP is not a marker for dorsal ipRGCs as shown previously in rat retina. Scale bars in a-c = 50 µm, d = 200 µm. This experiment was performed in a single animal.

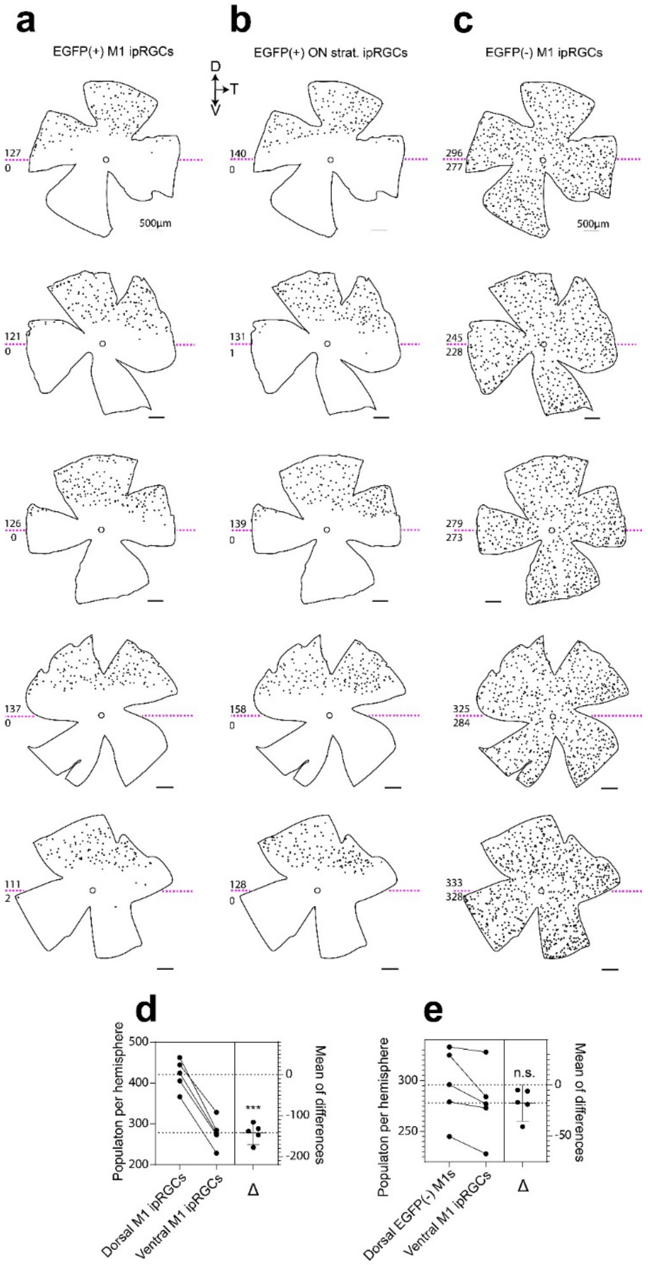

Supplementary Figure 5. Distribution maps of ipRGCs across the retina.
(a,b) Distribution of EGFP-positive M1 ipRGCs with dendrites stratiying in sublamina-a or **(b)** non-M1 ipRGCs with dendrites sublamina-b of the IPL in *GlyT2*^{Cre}*;Ai140* mice. **(c)** Distribution of EGFP-negative M1-ipRGCs with dendrites stratifying in sublamina-a in *GlyT2*^{Cre}*;Ai140* mouse line. Quantified values of dorsal versus ventral cells denoted above and below the dotted line transecting the optic nerve. **(d,e)** Population of M1 ipRGCs per retina in the dorsal vs ventral retina with (d) and without (e) the inclusion of EGFP-positive M1 ipRGCs. Mean of the differences (right) computed with a paired one-way ANOVA comparing number of cells (dorsal versus ventral; n=5 retina from n=3 animals; data are presented as mean values ± SEM; *** in d p=0.0002). Source data are provided as a source data file.

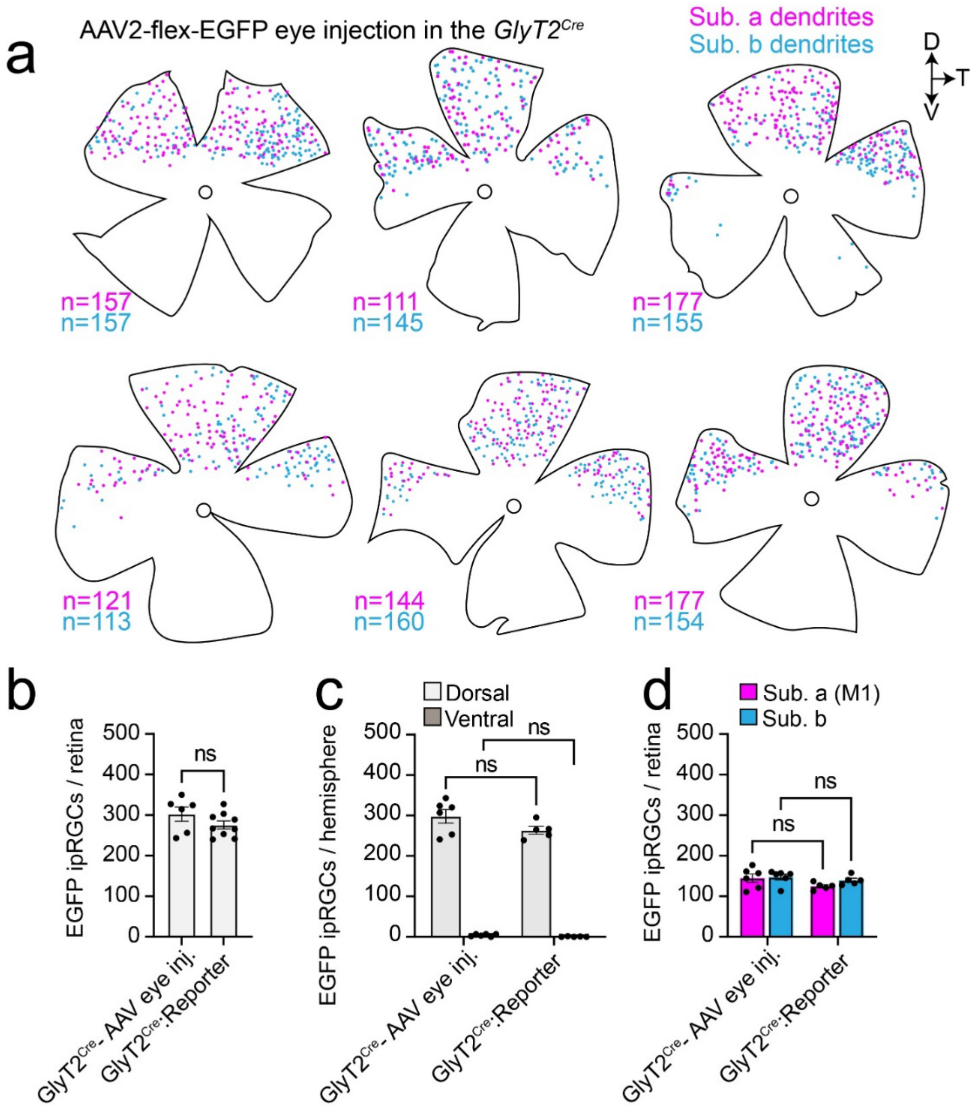

Supplementary Figure 6. Cre-dependent anterograde AAV labeling.
Unilateral stereotactic injections of *AAV2-FLEx-EGFP* into the eye of the *GlyT2^Cre^* mouse line labels *GlyT2^Cre^* ipRGCs. **(a)** Distribution maps of M1 ipRGCs with dendrites stratifying in sublamina-a or non-M1-ipRGCs with dendrites in sublamina-b. n = number of cells with dendritic stratification in sublamina-a or -b per retina. **(b,c)** Bar graphs of quantified ipRGCs per retina (b) and per hemisphere (c) labeled from eye injection versus labeled in the *GlyT2^Cre^;Ai140* mouse line. **(d)** Quantification of the number ipRGCs with dendrites stratifying in sublamina-a or sublamina-b in retinas labeled with eye injections versus those identified in the *GlyT2^Cre^;Ai140* (n=6 eye injection animals; n=7 *GlyT2^Cre^;Ai140* animals; data are presented as mean values ± SEM). Statistical significance assessed using one-way ANOVA. Source data are provided as a source data file.

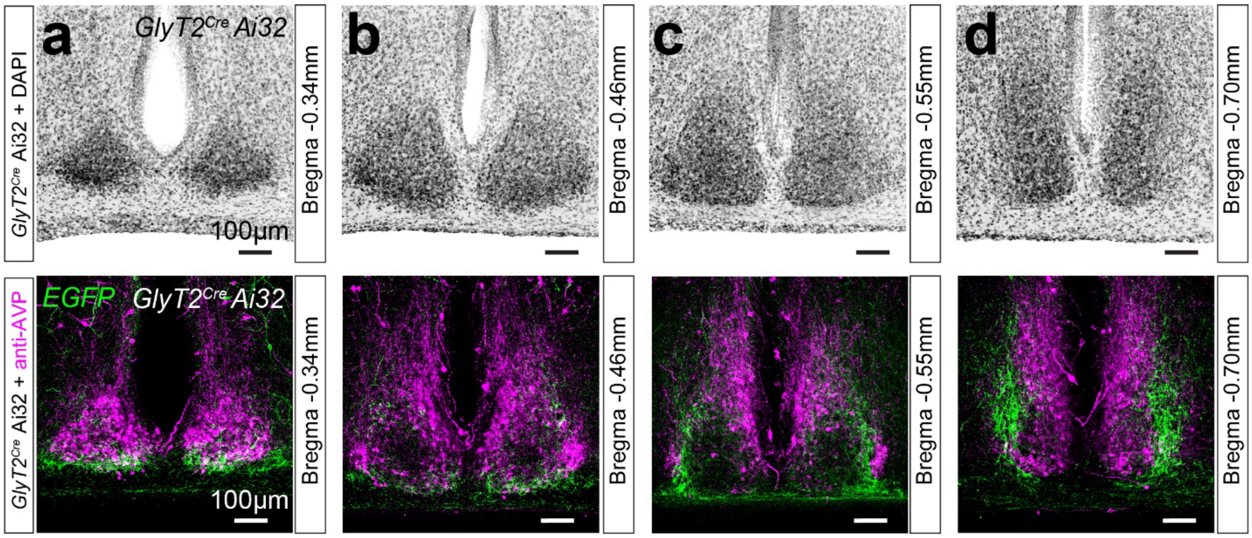

Supplementary Figure 7. Distinct anatomical areas of the SCN innervated by axons from *GlyT2*^{Cre} ipRGCs.

Coronal sections of the SCN of a *GlyT2*^{Cre}*;Ai32* mouse, presented anterior to posterior. EYFP-expressing *GlyT2*^{Cre} ipRGCs (bottom) innervate previously undescribed locations of the SCN labeled the outer core and lateral band, avoiding the SCN shell defined by AVP staining (bottom). DAPI (top) provides an anatomical reference. Scale bars: (a-d); 100 µm.

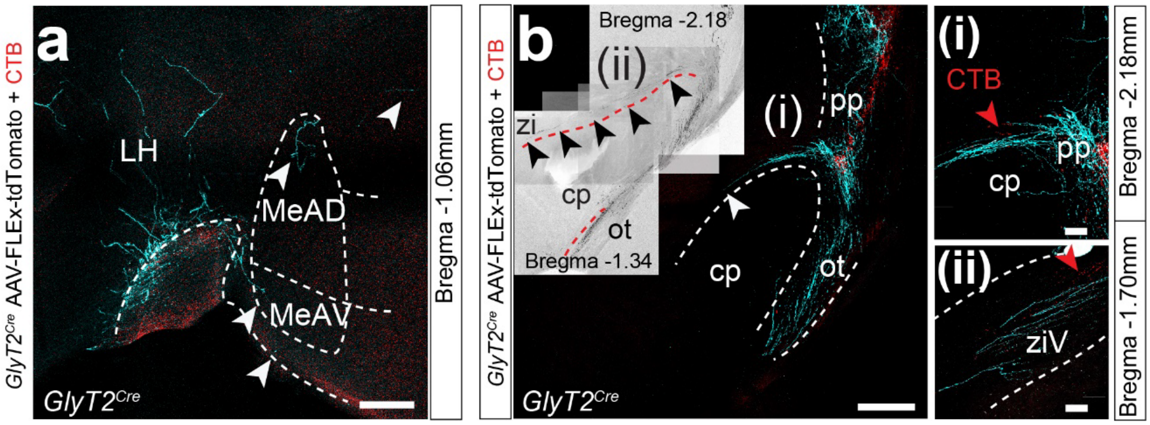

Supplementary Figure 8. Other sites of hypothalamic projections.
(a) Cre-dependent AAV (*AAV-FLEx-tdTomato*) eye injection in the *GlyT2*^{Cre} mouse line also labeled sparse projections in the lateral hypothalamus (LH) and in the anteroventral and anterodorsal edges of the medial amygdaloid nucleus (MeAD & MeAV). **(b)** At the level the rostral ventral lateral geniculate nucleus (vLGN), before entering the geniculate complex, *GlyT2*^{Cre} ipRGCs emerge from the optic tract and split at the peripeduncular nucleus (pp; i). A moderate number of ventromedial axons wrap around the cerebral peduncle (cp) and project nearly 1mm caudally along the ventral length of the zona incerta (ziV; i,ii = inlay from n; red arrow denotes presence of CTB). Scale bars: (a,b); 200 µm, (i,ii); 50 µm.

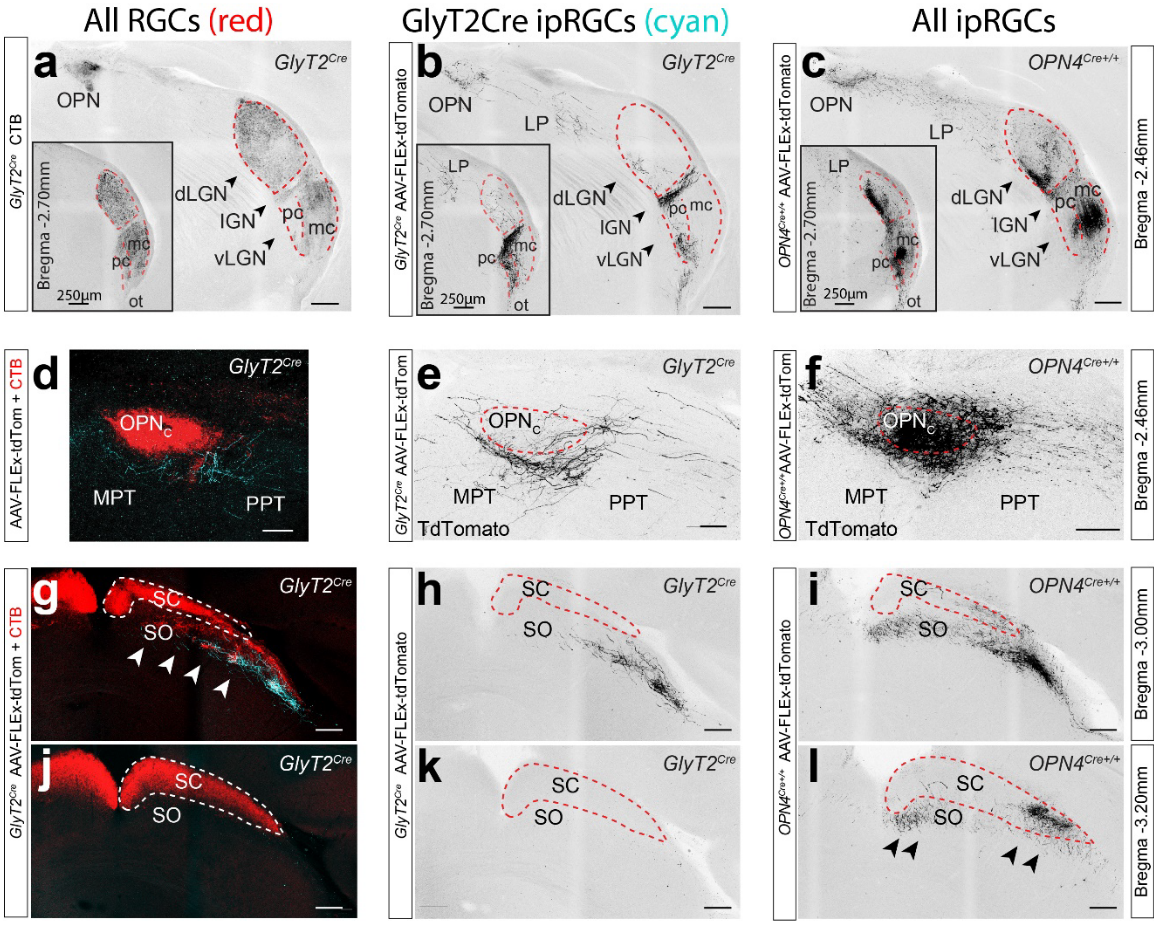

Supplementary Figure 9. Thalamic, pretectal, and SC ipRGC projections. Anterograde tracing of central projections in $GlyT2^{Cre}$ and $OPN4^{Cre}$ mouse lines following intravitreal eye injections with Cre-dependent AAV (*AAV-FLEx-tdTomato*). Cholera toxin subunit B (CTB), was included in eye injections to label all RGC axons (a,d,j). **(a-c)** Confocal images of coronal brain slice reveal that ipRGCs labeled in $GlyT2^{Cre}$ mice innervate the parvocellular (pc) division of the ventrolateral geniculate complex (vLGN) and the intergeniculate leaflet (IGL). **(b)** IpRGCs labeled in the $OPN4^{Cre}$ mouse line more broadly innervate the parvocellular (pc) and magnocellular (mc) divisions of the vLGN, IGL, and focal portions of the dorsal lateral geniculate nucleus (dLGN). **(c)** $GlyT2^{Cre}$ ipRGCs also form a plexus of terminals in the lateral posterior nucleus (LP). **(d-f)** Confocal images of the olivary pretectal nucleus (OPN) identify that $GlyT2^{Cre}$ ipRGCs innervate the ventral cup of the OPN shell, where ipRGCs labeled in the $OPN4^{Cre}$ mice project to both core and shell regions, similar to that observed by CTB labeling. Sparse innervation to neighboring pretectal structures such as medial pretectal (MPT) and posterior pretectal (PPT) areas are also observed. **(g-l)** $GlyT2^{Cre}$ ipRGCs innervate the superior colliculus (SC) **(h)**, like many ipRGCs and RGCs (g,i,j,l). These projections are sparse and localized to the superficial layer of the stratum opticum (SO; h), with very few projections to the central SC, unlike those in the $OPN4^{Cre}$ mouse line or with CTB labeling. Scale bars: (a-c,g-l); 250 µm, (d-f); 100 µm.

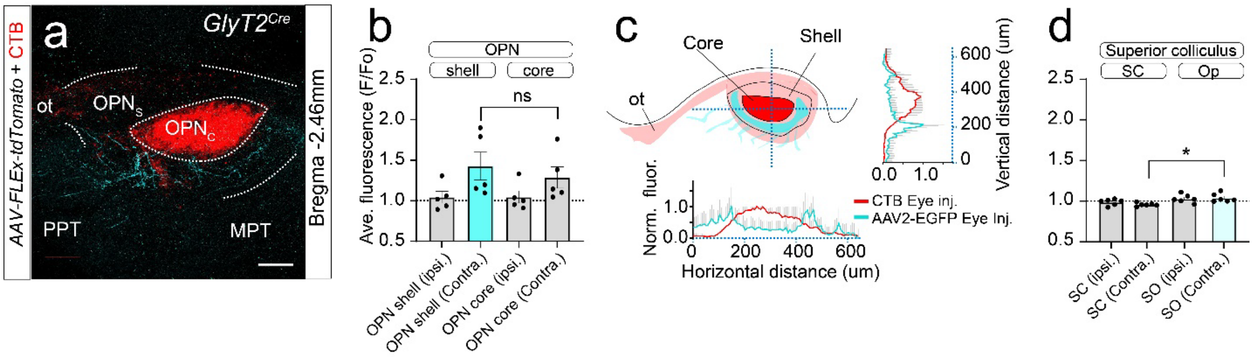

Supplementary Figure 10. *GlyT2^{Cre}* ipRGCs projections to the olivary pretectal nucleus.

(a) Confocal image of coronal brain slice following a *Cre*-dependent eye injection in a *GlyT2^{Cre}* mouse reveals sparse innervation of the ventral shell of the OPN, avoiding the OPN core and dorsal hemisphere of the OPN shell. Scale bar = 100 μm **(b)** Quantification of the OPN innervation pattern using normalized fluorescence values (F/Fo) of OPN core and shell suggest sparse innervation for both the ipsilateral and contra-lateral hemispheres (F/Fo<1.5; data are presented as mean values ± SEM). **(c)** Normalized cross-sectional fluorescence profiles plotted through the axis of the contralateral OPN (dotted line) for *AAV2-FLEx-EGFP* vs. CTB (eye injections in the *GlyT2^{Cre}* mouse line. Profiles illustrate the pattern of *GlyT2^{Cre}* ipRGC innervation to the ventral shell OPN avoiding the OPN core and dorsal shell. **(d)** Quantification of the sparse innervation pattern to the superior colliculus (SC) and stratum opticum (SO) using normalized fluorescence values (F/Fo) for both the ipsilateral and contra-lateral hemispheres (F/Fo<1.5). Quantification performed in mice following unilateral eye injection of *AAV2-FLEx-EGFP* (n=5 animals; data are presented as mean values ± SEM; statistical significance was assessed using one-way ANOVA; *p = 0.0332). Source data are provided as a source data file.

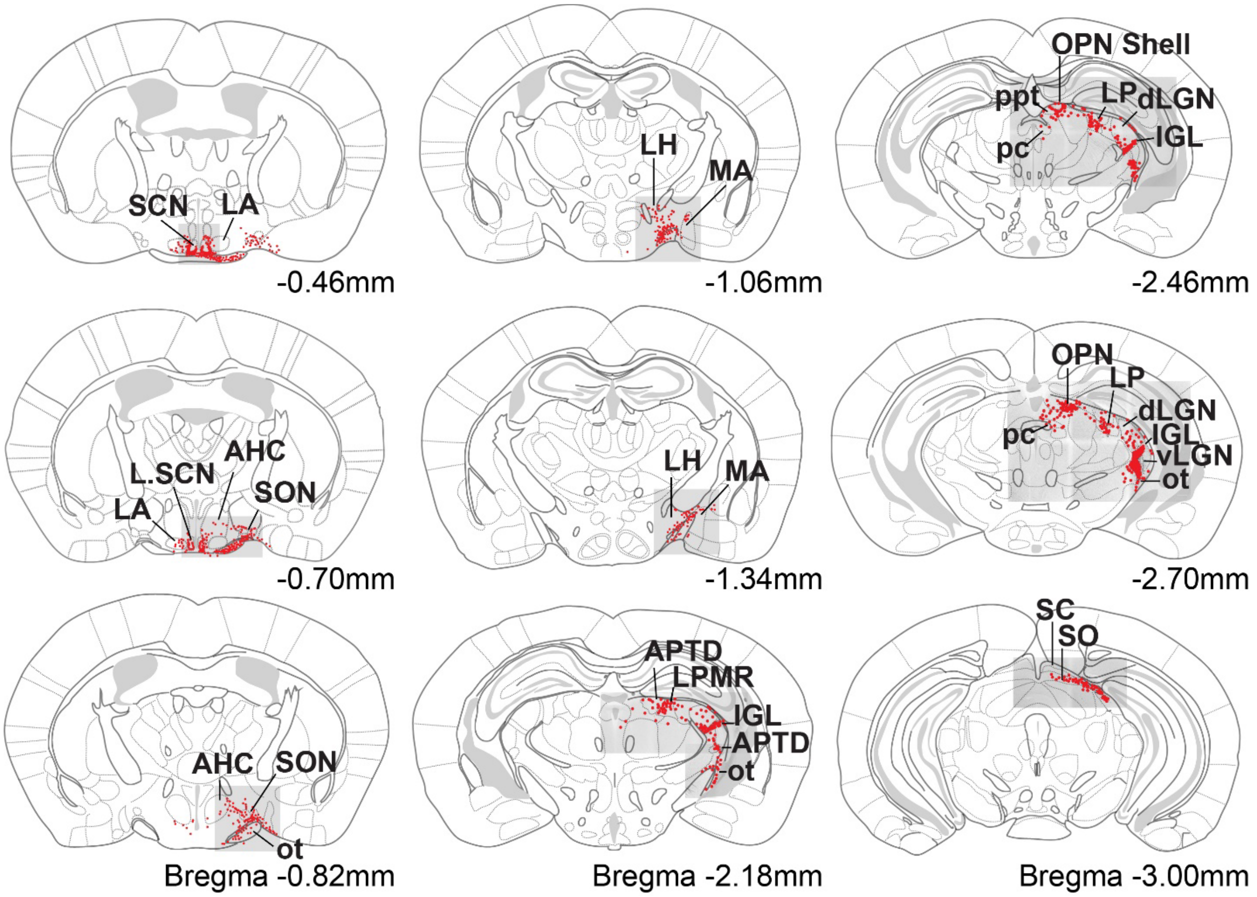

Supplementary Figure 11. Summary of ipRGC central projections in the *GlyT2*^{Cre} mouse

Graphical atlas maps (red: axons and terminals) drawn from the brain slices of *GlyT2*^{Cre} mice using the Paxinos & Franklin The Mouse Brain In Stereotaxic Coordinates (5th addition Figures; 35,37,38,40,42,49, 51, 53, 56). Suprachiasmatic nucleus (SCN) supraoptic nucleus (SON), optic tract (ot), lateral anterior hypothalamic (LA), anterior hypothalamic central (AHC), lateral hypothalamus (LH), Medial amygdaloid nucleus (MA) superior colliculus (SC), stratum opticum (SO), posterior pretectal area (PPT), olivary pretectal nucleus (OPN), ventrolateral geniculate complex (vLGN), intergeniculate leaflet (IGL), lateral posterior nucleus (LP), dorsal lateral geniculate nucleus (dLGN).

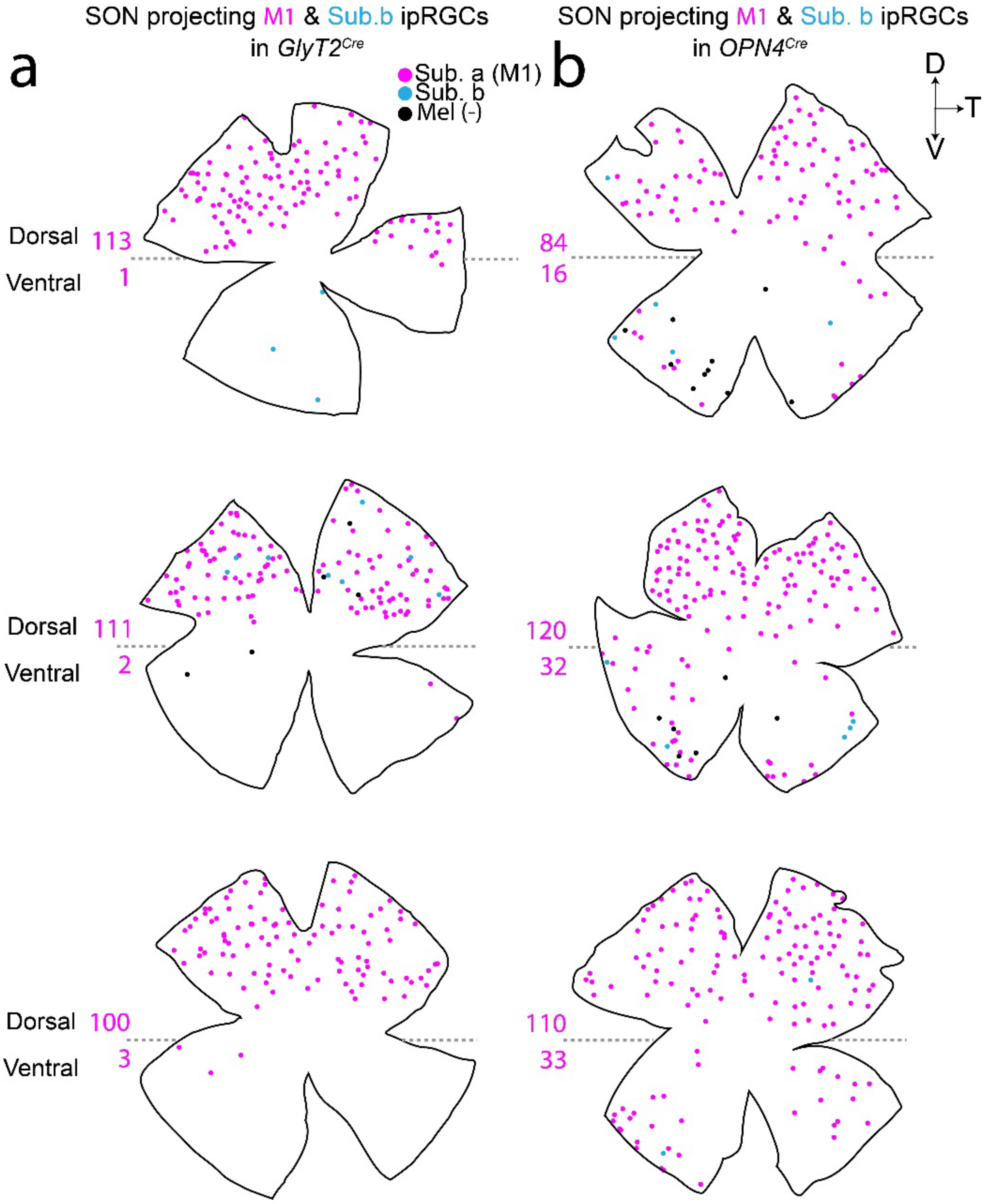

Supplementary Figure 12. Retinal maps of SON projecting ipRGCs.
(a,b) Retina distribution maps of cells label in the retina following stereotactic *Cre*-dependent retroAAV injection to the SON in $Glyt2^{Cre}$ (a) and $OPN4^{Cre}$ (b) mice. Numerical values represent the quantified number of ipRGCs in the dorsal (top) and ventral retinal hemisphere divided through the optic nerve. Source data are provided as a source data file.

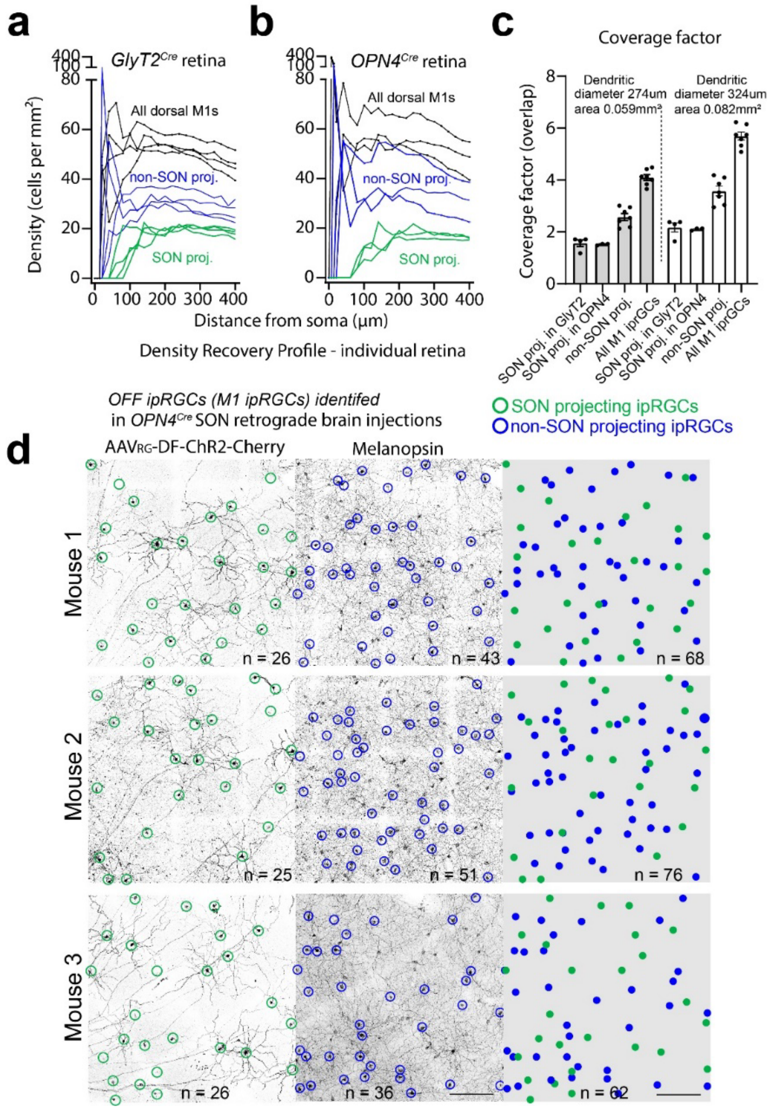

Supplementary Figure 13. Stereotactic SON injection identifies M1 *GlyT2^Cre^* ipRGCs
(a,b) Density recovery profile (per retina) of dorsal M1 ipRGCs labeled by central injection into the SON in *GlyT2^Cre^* (a) and *OPN4^Cre^* (b) mice. Density recovery profiles display density of neighboring M1 ipRGC cell bodies at distances (µm) from each soma for All M1 ipRGCs, non-SON projecting M1 ipRGCs, and SON-projection M1 ipRGCs. M1 ipRGCs were identified morphologically. SON-projecting M1 ipRGCs = labeled from retrograde injection, non-SON projecting M1 ipRGCs = M1 ipRGCs not labeled from retrograde injection, All dorsal M1 ipRGCs = SON + non-SON projecting M1 ipRGCs. **(c)** Coverage factor illustrating the proportion of dendritic overlap calculated from the average diameter of *GlyT2^Cre^* M1 ipRGCs measured from either Neurobiotin fills (left) or calculated from the average diameter of M1 ipRGCs reported by Berson et al. 2002 (right). **(d)** Dorsal retina of M1 ipRGCs labeled by retrograde SON injections in *OPN4^Cre^* mice. M1 ipRGCs not labeled by central injection and overlap of SON and non-SON projecting M1 ipRGCs in the dorsal retina. N = 3 retina for each strain. Area = 1mm^2. Values are mean ± SEM. Scale bars: (d); 200 µm, (i,ii); 200 µm. Source data are provided as a source data file.

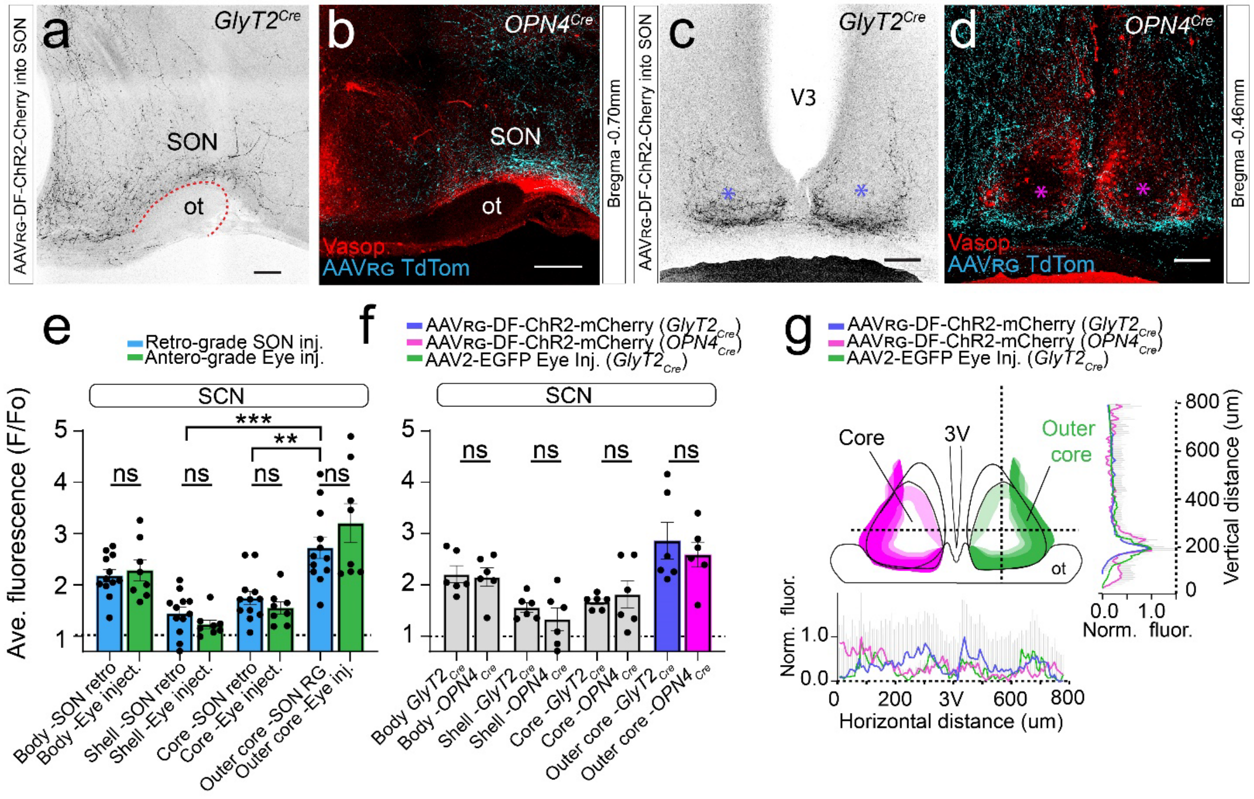

Supplementary Figure 14. Collateral SCN innervation of retro-labeled SON ipRGCs. (a-d) Injections of Cre-dependent retroAAV (*AAVRG-DF-ChR2-mCherry*) into the SON of *GlyT2*^{Cre} and *OPN4*^{Cre} mice. Innervation pattern of collateral projections labeled by anterograde eye injection (*AAV2-FLEx-EGFP*) in *GlyT2*^{Cre} mice. **(c, d)** Collateral innervation is observed in confocal images of coronal SCN brain slice (200um). **(e)** Normalized fluorescence values (F/Fo) of the entire SCN (body), SCN core, shell and SCN outer core in *GlyT2*^{Cre} and *OPN4*^{Cre} animals that received retrograde SON AAV injection compared with *GlyT2*^{Cre} animas that received anterograde eye injections (adopted from Figure 5 for comparison purposes, data are presented as mean values ± SEM; ***p=0.0002, **p=0.0024). **(f)** Normalized fluorescence values (F/Fo) of the SCN body, SCN core, Shell and SCN outer core in *GlyT2*^{Cre} and *OPN4*^{Cre} animals that received retrograde SON AAV injections (data are presented as mean values ± SEM). **(g)** Illustration (left) and normalized cross-sectional fluorescence profiles plotted through the horizontal and ventral axis of SCN (dotted line) for *Cre*-dependent retroAAV in in *GlyT2*^{Cre} or *OPN4*^{Cre} and *AAV2-FLEx-EGFP* in *GlyT2*^{Cre}. Profiles illustrate the outer core pattern of the SCN is retained when labeling the SON ipRGC collaterals in either the *GlyT2*^{Cre} or *OPN4*^{Cre} mouse lines. SON retroAAV in *GlyT2*^{Cre}; n= 5, SON retroAAV in *OPN4*^{Cre}; n= 6. *AAV2-FLEx-EGFP* in *GlyT2*^{Cre} mice, n= 6 animals. Statistical significance was assessed using one-way ANOVA. Scale bar = (a,b); 250 µm, (c,d); 100 µm. Source data are provided as a source data file.

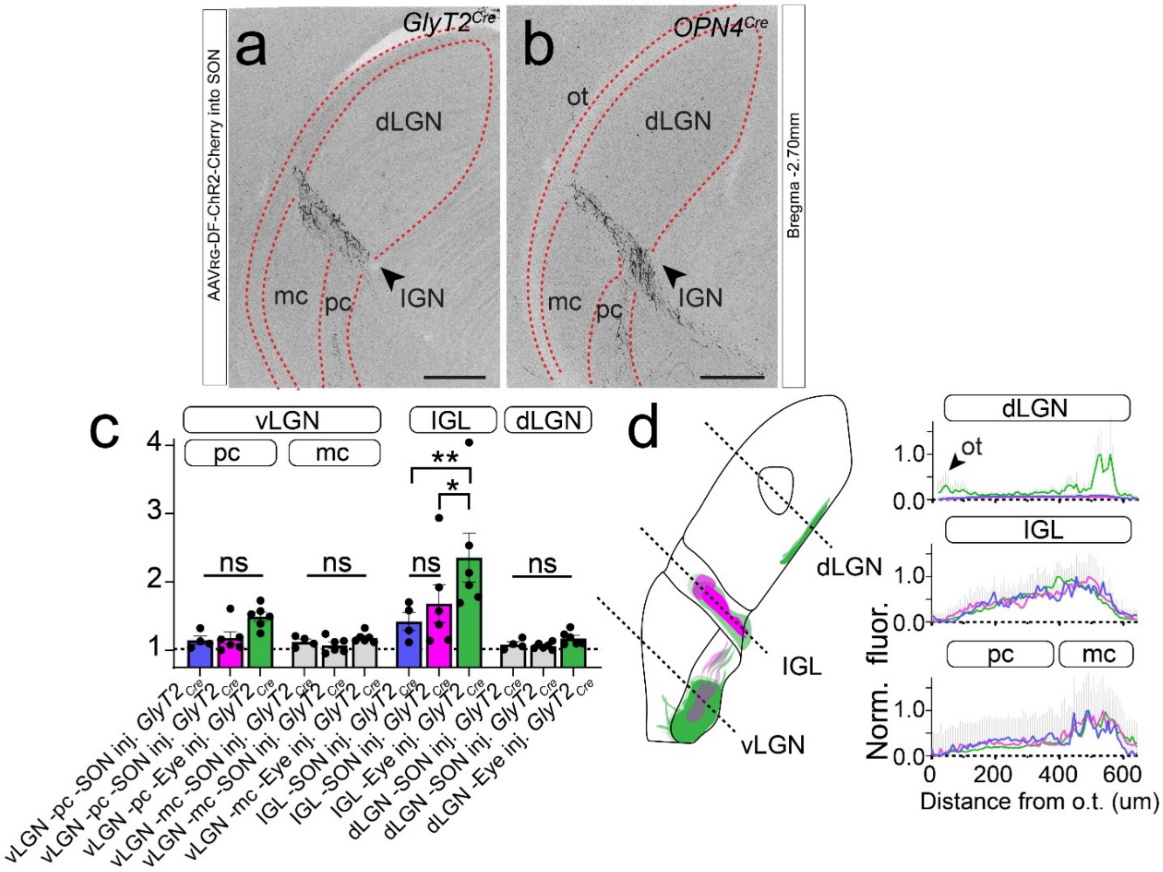

Supplementary Figure 15. SON ipRGCs have unique collateral innervation of the IGL & vLGN.

(a-b) Confocal micrographs of the LGN following injections of *Cre*-dependent retroAAV (*AAV_{RG}-DF-ChR2-mCherry*) into the supraoptic nucleus (SON) of *GlyT2^{Cre}* and *OPN4^{Cre}* mice. Innervation pattern of collateral projections compared with projections labeled by anterograde eye injection (*AAV2-FLEx-EGFP*) in *GlyT2^{Cre}* mouse. Collateral innervation is observed in intergeniculate leaflet (IGL) and parvocellular (pc) division of the vLGN in both *GlyT2^{Cre}* and *OPN4^{Cre}* mice. Innervation of the dLGN is absent. **(c)** Normalized fluorescence values (F/Fo) of the pc and magnocellular (mc) regions of the vLGN, IGL, and dLGN (contra-lateral hemispheres) in *GlyT2^{Cre}* and *OPN4^{Cre}* animals that received retrograde SON AAV injection compared with *GlyT2^{Cre}* animals that received anterograde eye injections (adopted from Figure 5 for comparison purposes; data are presented as mean values ± SEM; **p=0.0024; *p=0.0246). **(d)** Normalized cross-sectional fluorescence profiles plotted through the axis of the contralateral vLGN, IGL, and dLGN (dotted line) for *Cre*-dependent retroAAV SON injected *GlyT2^{Cre}* or *OPN4^{Cre}* and anterograde AAV eye injected in *GlyT2^{Cre}*. Quantification illustrates no difference in innervation between the SON ipRGCs labeled in the *GlyT2^{Cre}* or *OPN4^{Cre}* mice. However, pattern of SON ipRGC innervation to the pc region of the vLGN and medial predominance of the IGL, is reduced and innervation to the dLGN is absent compared to the anterograde labeled ipRGCs in the eye injected *GlyT2^{Cre}*. SON retroAAV in *GlyT2^{Cre}*; n= 4, SON retroAAV in OPN4^{Cre}; n= 6. *AAV2-FLEx-EGFP* in *GlyT2^{Cre}*; n= 6 animals. Statistical significance was assessed using one-way ANOVA. Scale bar = (a,b); 250 μm. Source data are provided as a source data file.

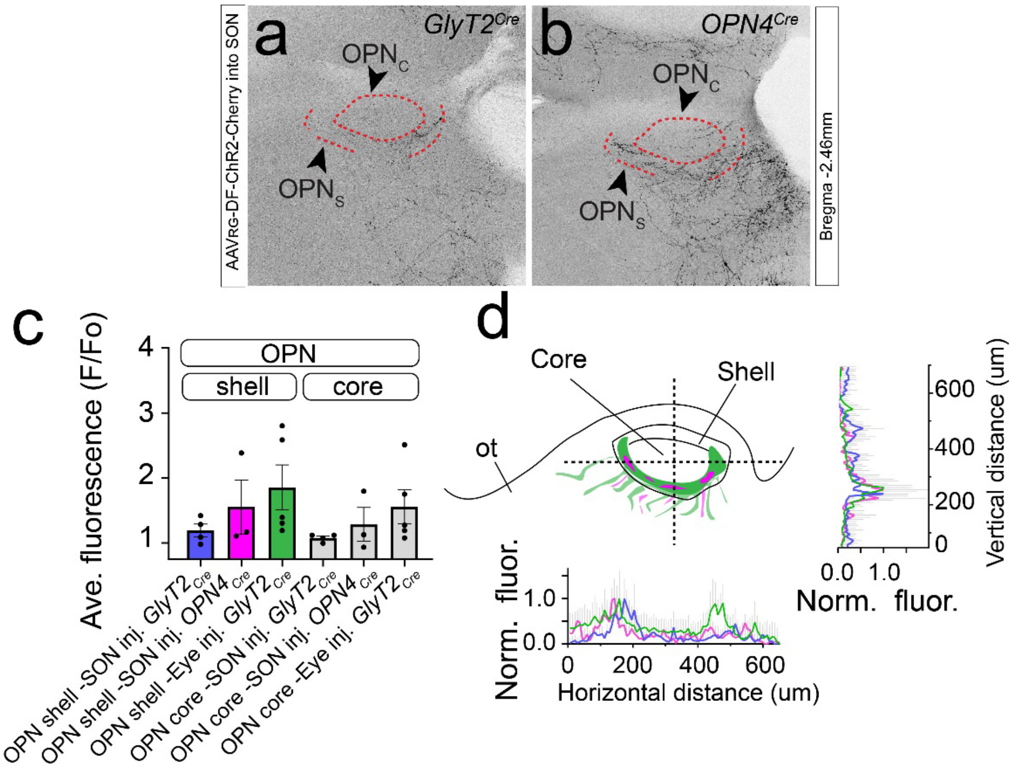

Supplementary Figure 16. SON ipRGCs have sparse collateral innervation of the olivary pretectal nucleus (OPN).

(a-b) Confocal micrographs of the OPN following focal injections of Cre-dependent retroAAV (*AAVRG-DF-ChR2-mCherry*) into the supraoptic nucleus (SON) of *GlyT2*^{Cre} and *OPN4*^{Cre} mice. Sparse collateral innervation is observed in the shell of the OPN in in both mice. **(c)** Normalized fluorescence values (F/Fo) of the OPN shell and core (contra-lateral hemispheres) in *GlyT2*^{Cre} and *OPN4*^{Cre} animals that received retrograde SON AAV injection compared with *GlyT2*^{Cre} animals that received anterograde eye injections (adopted from Supplementary 9 for comparison purposes; data are presented as mean values ± SEM). **(d)** Normalized cross-sectional fluorescence profiles plotted through the horizontal and vertical axis of the contralateral OPN (dotted line) for *Cre*-dependent retroAAV SON injected *GlyT2*^{Cre} or *OPN4*^{Cre} and anterograde AAV eye injected in *GlyT2*^{Cre} mice. Quantification and profiles illustrate that the pattern of SON ipRGC innervation to the OPN retains the ventral shell predominance but is reduced or absent (3/6 *OPN4*^{Cre} animals) compared to the already sparse anterograde labeled ipRGCs in the eye injected *GlyT2*^{Cre}. SON retroAAV in *GlyT2*^{Cre}; n= 4 animals, SON retroAAV in *OPN4*^{Cre}; n= 6 animals. *AAV2-FLEx-EGFP* in *GlyT2*^{Cre}; n= 6 animals. Statistical significance was assessed using one-way ANOVA. Scale bar = (a,b); 250 µm. Source data are provided as a source data file.

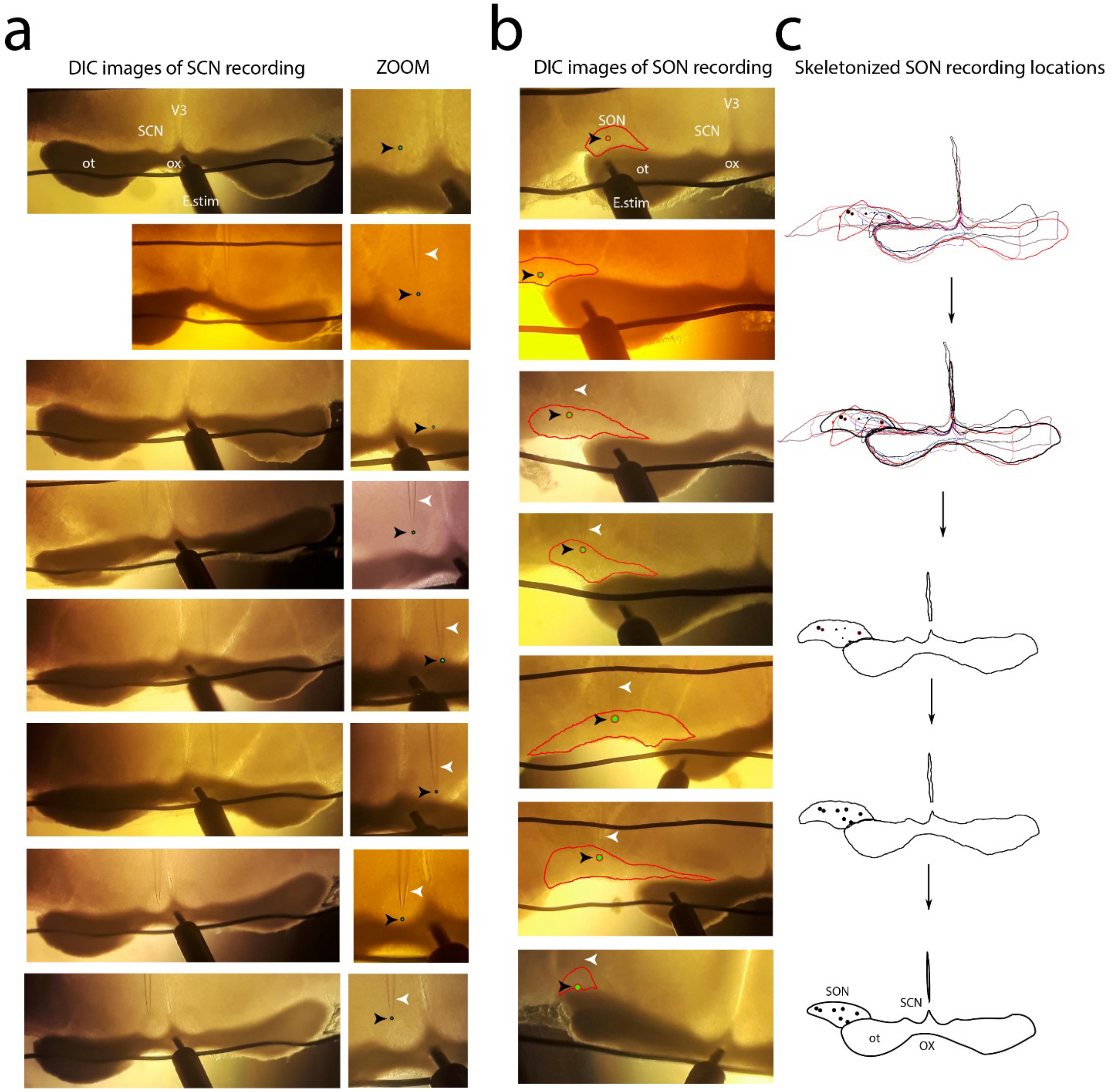

Supplementary Figure 17. DIC images of SCN and SON recordings
(a,b) DIC images displaying the location of photo-responsive SCN (a) and SON (b) neurons in the *GlyT2*^{Cre}*;Ai32* mouse. Black arrowhead; cell body of the photo-responsive cell, white arrowhead; glass pipet of the recording electrode. E.stim = electrical stimulator used to confirm photo-responsive cells were retinorecipient. **(c)** Skeletonized localization of photo-responsive SON cells.

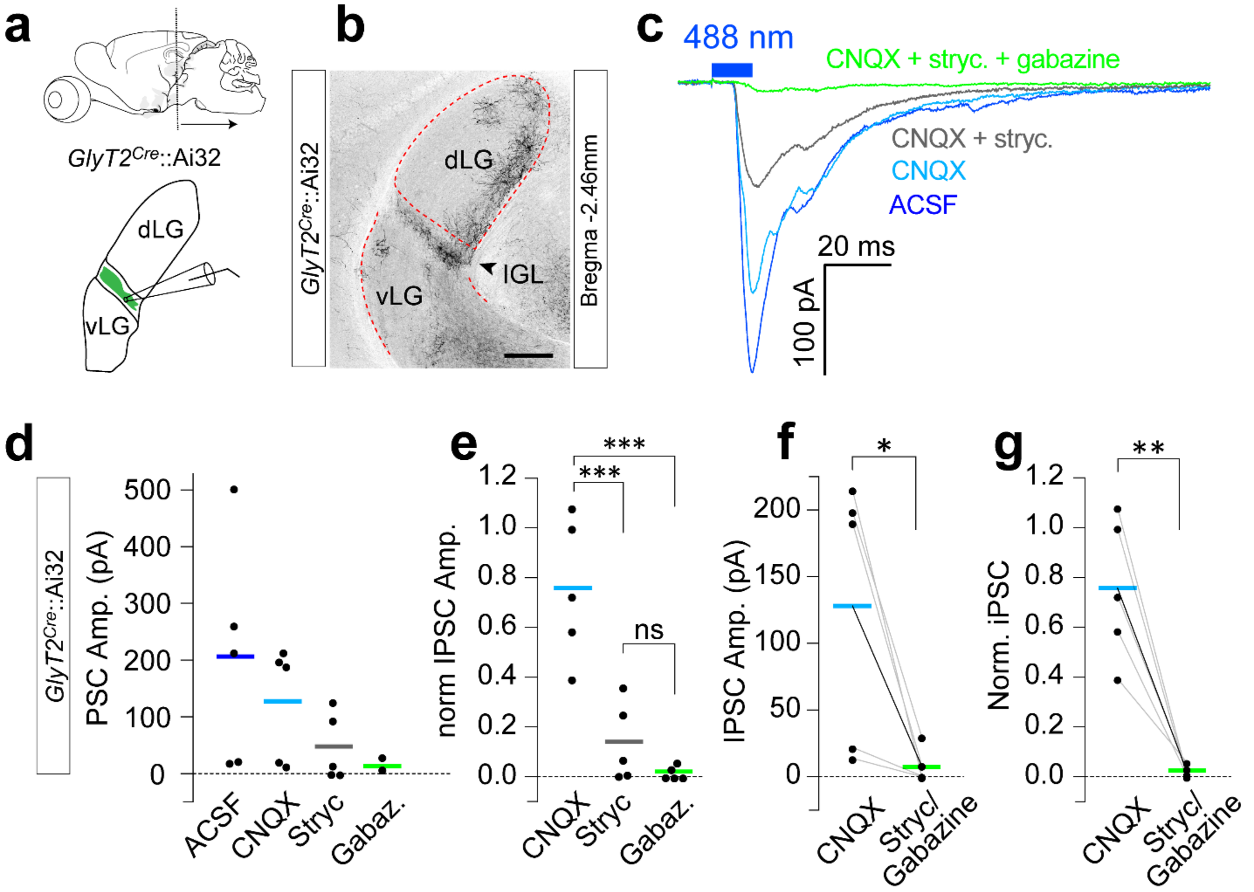

Supplementary Figure 18. Inhibitory inputs to the IGL in *GlyT2^{Cre};Ai32* brain slice. **(a)** Coronal sections were used for brain slice recordings in the IGL of the *GlyT2Cre;Ai32* mouse. **(b)** Confocal image of the ventral lateral geniculate nucleus vLGN, IGL, and the dLGN illustrating EYFP expression in *GlyT2^{Cre};Ai32* brain. Scale bar = 250 µm **(c)** Whole cell voltage clamp recording traces of photo-stimulation under Control, 20 µM, 0.5 µM strychnine + CNQX, and 3 µM gabazine + strychnine + CNQX. **(d,e)** Photo-response amplitude (d) and normalized photo-response amplitude (e) of PSCs under antogonist conditions (***left/middle p=0.0005; left/right p=0.001; middle/right ns p=0.3285; one-way ANOVA with Holm-Sidak's test). **(f,g)** Photo-response amplitude (f; *p=0.0477; two-tailed paired t-test) and normalized photo-response amplitude (g; ** p=0.0056; two-tailed paired t-test) in CNQX before and after the addition of gabazine + strychnine.

Supplementary Table 1: Antibodies and immunostaining

Antibodies/Dyes	Concentration	Duration	Source	Identifier	RRID
Cholera toxin subunit B, Alexa Fluor 488 conjugate	1-μl 0.5% eye injection	7 days	ThermoFisher	C34775	
Rabbit anti-melanopsin	1:2000	Retina - 3days - (rt)	ATSBio	AB-N39	AB_1608077
Chicken anti-mCherry	1:1000	Retina - 3days - (rt)	Abcam	ab205402	AB2722769
	1:1000	Brain slice - overnight (rt)			AB2722769
Goat anti-GFP	1:1000	Retina - 3days - (rt)	Abcam	ab5450	AB_304897
	1:1000	Brain slice - overnight (rt)			AB_304897
Chicken anti-GFP	1:2000	Retina - 3days - (rt)	Aves labs	GFP-1020	AB_2307313
	1:2000	Brain slice - overnight (rt)			AB_2307313
Goat anti-Cholera Toxin subunit B	1:4000	Brain slice - over night (rt)	List Labs	#703	AB_10013220
Rabbit anti-vasopressin	1:5000	Brain slice - overnight (rt)	Immunostar	#20069	AB_572219
Rabbit anti-Opsin Blue	1:1000	Retina - 3days - (rt)	Millipore	ab5407	AB_177457
Mouse anti-Neurofilament H (SMI-32)	1:1000	Retina - 3days - (rt)	BioLegend	ab509997	AB_509997
Guinea Pig anti-RBPMS	1:2000	Retina - 3days - (rt)	Phosphosolutions	1832-RBPMS	AB_2492226
Donkey anti-rabbit Alexa Fluor 647	1:2000	Overnight - (rt)	Jackson Immuno Research	711-545-152	AB_2313584
Donkey anti-goat Alexa Fluor 488	1:1000	Overnight - (rt)	Jackson Immuno Research	705-545-147	AB_2336933
Donkey anti-chicken Cy3	1:2000	Overnight - (rt)	Jackson Immuno Research	703-165-155	AB_2340363
Donkey anti-chicken Alexa Fluor 488	1:2000	Overnight - (rt)	Jackson Immuno Research	703-545-155	AB_2340375
Donkey anti-mouse Alexa Fluor 488	1:2000	Overnight - (rt)	Jackson Immuno Research	715-545-151	AB_2341099
Donkey anti-mouse Alexa Fluor 594	1:2000	Overnight - (rt)	Jackson Immuno Research	715-585-151	AB_2340855
Donkey anti-mouse Alexa Fluor 647	1:2000	Overnight - (rt)	Jackson Immuno Research	715-605-151	AB_2340862
Donkey anti-Guinea Pig Alexa Fluor 488	1:2000	Overnight - (rt)	Jackson Immuno Research	706-545-148	AB_2340472
Donkey anti-Guinea Pig Alexa Fluor 647	1:2000	Overnight - (rt)	Jackson Immuno Research	715-605-148	AB_2340476

Supplementary Table 2: Coverage Factors for Various ipRGC cells from GlyT2 and OPN4 mice using random intercept mixed effects models.

	Area from Fills (0.082mm ^{2})		Area from Berson (0.059mm ^{2})		
Cell Type	GlyT2 [a]	OPN4 [a]	GlyT2 [a]	OPN4 [a]	p-value[b]
SON M1 ipRGCs	2.17 (0.247)	2.1 (0.259)	1.56 (0.178)	1.51 (0.186)	0.851
Non-SON M1 ipRGCs	3.59 (0.247)	3.55 (0.259)	2.58 (0.178)	2.56 (0.186)	0.926
All M1 ipRGCs	5.74 (0.247)	5.63 (0.259)	4.13 (0.178)	4.05 (0.186)	0.762

[a] Values written as Mean (SEM).
[b] P-values comparing means for OPN4 vs.GlyT2 coverages from two-sided t-tests using Satterthwaite's method.

Supplementary Table 3: Box and Whisker parameters from Figure 2.

Fig. 2c		Sub. a GlyT2Cre	Sub. a Brn3bzDTA	Sub. b GlyT2Cre	Sub. b (small) GlyT2Cre
Branches	Number of values	11	8	11	5
	Minimum	20	9	60	36
	25% Percentile	30	26.25	74	76.5
	Median	48	37	101	129
	75% Percentile	65	60.5	112	167.5
	Maximum	79	74	169	176
	Range	59	65	109	140
	Mean	47.82	40.25	99.45	123.4
	Std. Deviation	18.9	21.46	29.65	54.19
	Std. Error of Mean	5.7	7.587	8.939	24.23
Junctions	Number of values	11	8	11	5
	Minimum	10	3	30	16
	25% Percentile	15	11.75	36	36.5
	Median	22	19	51	64
	75% Percentile	33	32.75	56	84
	Maximum	38	38	84	88
	Range	28	35	54	72
	Mean	23.18	20.38	49.27	61
	Std. Deviation	8.953	11.98	14.88	28.02
	Std. Error of Mean	2.7	4.234	4.487	12.53
Endpoints	Number of values	11	8	11	5
	Minimum	10	9	28	22
	25% Percentile	14	11.5	33	34
	Median	22	16	39	56
	75% Percentile	24	20.5	43	64.5
	Maximum	40	25	65	68
	Range	30	16	37	46
	Mean	21.73	16.25	40.27	50.6
	Std. Deviation	9.655	5.312	9.951	17.88
	Std. Error of Mean	2.911	1.878	3	7.997
Fig. 2d					
Soma diameter	Number of values	11	8	11	5
	Minimum	11.55	12.02	13.41	14.72
	25% Percentile	12.99	12.67	15.19	16.23
	Median	13.46	13.96	16.04	18.28
	75% Percentile	15.6	15.61	17.1	20.47
	Maximum	16.65	16.63	19.17	20.8
	Range	5.099	4.61	5.762	6.079
	Mean	13.99	14.14	16.2	18.33
	Std. Deviation	1.609	1.66	1.664	2.385
	Std. Error of Mean	0.4851	0.5868	0.5018	1.067
Fig. 2e					
Dendritic diameter	Number of values	11	8	11	5
	Minimum	193.3	236	259.3	143.2
	25% Percentile	306.2	284.2	341.1	146.3
	Median	341.6	333.8	363.8	173.4
	75% Percentile	363.1	399.1	418.2	245
	Maximum	423.5	442.7	467.2	270.5
	Range	230.3	206.7	208	127.3
	Mean	324.5	337.5	374.2	191.2
	Std. Deviation	59.8	68.48	60.24	53.55
	Std. Error of Mean	18.03	24.21	18.16	23.95
