# Supplementary material for: A melanopsin ganglion cell subtype forms a dorsal retinal mosaic projecting to the supraoptic nucleus
Source: Nat Commun. 2023 Mar 17;14:1492. doi: 10.1038/s41467-023-36955-6 (PMC10023714; doi:10.1038/s41467-023-36955-6)
Supplement: Supplementary file 3 — Reporting Summary [file 41467_2023_36955_MOESM3_ESM.pdf]

nature portfolio

Corresponding author(s): Benjamin Sivyer
Last updated by author(s): Feb 2, 2023

Reporting Summary

Nature Portfolio wishes to improve the reproducibility of the work that we publish. This form provides structure for consistency and transparency in reporting. For further information on Nature Portfolio policies, see our Editorial Policies and the Editorial Policy Checklist.

Statistics

For all statistical analyses, confirm that the following items are present in the figure legend, table legend, main text, or Methods section.

n/a	Confirmed	
☐	☒	The exact sample size (*n*) for each experimental group/condition, given as a discrete number and unit of measurement
☐	☒	A statement on whether measurements were taken from distinct samples or whether the same sample was measured repeatedly
☐	☐	The statistical test(s) used AND whether they are one- or two-sided<br>*Only common tests should be described solely by name; describe more complex techniques in the Methods section.*
☐	☒	A description of all covariates tested
☐	☒	A description of any assumptions or corrections, such as tests of normality and adjustment for multiple comparisons
☐	☒	A full description of the statistical parameters including central tendency (e.g. means) or other basic estimates (e.g. regression coefficient) AND variation (e.g. standard deviation) or associated estimates of uncertainty (e.g. confidence intervals)
☐	☒	For null hypothesis testing, the test statistic (e.g. *F*, *t*, *r*) with confidence intervals, effect sizes, degrees of freedom and *P* value noted<br>*Give P values as exact values whenever suitable.*
☒	☐	For Bayesian analysis, information on the choice of priors and Markov chain Monte Carlo settings
☒	☐	For hierarchical and complex designs, identification of the appropriate level for tests and full reporting of outcomes
☒	☐	Estimates of effect sizes (e.g. Cohen's *d*, Pearson's *r*), indicating how they were calculated

Our web collection on statistics for biologists contains articles on many of the points above.

Software and code

Policy information about availability of computer code

Data collection	Confocal microscopy z-stacks were captured and tiles were stitched together in Leica LAS X Life Sciences software (version 3.7.4.23463). Patch clamp recordings were acquired using Axograph (version 1.7.4) or Patchmaster (v2x73.5 & v2x90.5; HEKA Elektronik Dr.Schulze GmbH). Visual stimuli used in recordings were generated using pyStim (Sivyer et al. 2019) which is available on Github (https://github.com/SivyerLab/pyStim).
Data analysis	Electrophysiology data was analyzed in Axograph (Version 1.7.4) and Igor Pro (version 6.37 and 8.04) Values of electrophysiology data taken from Axograph were quantified in Graphpad Prism (version 9) Confocal microscopy images were analyzed using ImageJ (Version 1.53q). Nearest neighbor distances were calculated using an ImageJ plugin https://icme.hpc.msstate.edu/mediawiki/index.php/Nearest_Neighbor_Distances_Calculation_with_ImageJ.html. Modeled randomization of points was produced using an ImageJ plugin (https://imagej.nih.gov/ij/macros/DrawRandomDots.txt).

For manuscripts utilizing custom algorithms or software that are central to the research but not yet described in published literature, software must be made available to editors and reviewers. We strongly encourage code deposition in a community repository (e.g. GitHub). See the Nature Portfolio guidelines for submitting code & software for further information.

Data

Policy information about availability of data

All manuscripts must include a data availability statement. This statement should provide the following information, where applicable:

- Accession codes, unique identifiers, or web links for publicly available datasets
- A description of any restrictions on data availability
- For clinical datasets or third party data, please ensure that the statement adheres to our policy

All data generated or analyzed during this study are included in this published article (and its supplementary information files). Source data are provided with this paper.

Human research participants

Policy information about studies involving human research participants and Sex and Gender in Research.

Reporting on sex and gender	N/A. Animal study.
Population characteristics	N/A. Animal study.
Recruitment	N/A. Animal study.
Ethics oversight	N/A. Animal study.

Note that full information on the approval of the study protocol must also be provided in the manuscript.

Field-specific reporting

Please select the one below that is the best fit for your research. If you are not sure, read the appropriate sections before making your selection.

- [x] Life sciences
- [ ] Behavioural & social sciences
- [ ] Ecological, evolutionary & environmental sciences

For a reference copy of the document with all sections, see nature.com/documents/nr-reporting-summary-flat.pdf

Life sciences study design

All studies must disclose on these points even when the disclosure is negative.

Sample size	No statistical methods were used to predetermine sample sizes and sample sizes arose by using the minimum number of animals to produce a statistically significant and reproducible result.
Data exclusions	For animals crossed with a fluorescent reporter, no data was excluded. For brain injections into the SON, n = 2 animals with minimal retro-labeled cells in the retina, were excluded from the analysis. Given the depth and size of the SON we attribute this to a failed central injection. Brain recordings were excluded if they did not produce a photo-inducible current. In the SCN recordings: n = 23 cells were excluded. In the IGL, n = 53 cells were excluded. In the SON, n = 12 cells were excluded. For the stereotactic eye injections 9 animals were unilaterally injected. 6 animals exhibit dense expression and brains were collected and sectioned. The other 3 animals were excluded because they exhibited poor eye expression and would make central projections challenging to interpret. Brain sections were cut at 200um and brain regions were localized using DAPI staining, immuno-staining of arginine vasopressin, and referenced to the Franklin & Paxinos Mouse Brain Coordinate Atlas. Brain slices where structures could not be confidently identified, were damaged, or were sectioned irregularly (given the 200um thickness) were not included in the quantification (SCN & SON; N=2/6 not included. OPN; N = 1/6 not included.
Replication	All attempts at replication were successful and are described in detail in the figure legends. Where the data is presented as representative examples we have provided details of the replication numbers in the 'Statistics and Reproducibility' section of the methods: "Statistical analysis is indicated in the figure legends or in tables where applicable. For data where single or representative micrographs are shown, we repeated each experiment independently with similar results as follows: Figures 1a,e=14 animals; 1c,d=7 animals; 3a,b=14 animals; 3c=1 animal; 4b,c=21 animals; 5 a,b=4 animals; 5c=1 animal; 5f=4 animals; 5i= 4 animals; 5j= 6 animals; 5m=4 animals; 6c, f=3 animals; 7c,d=3 animals; 8c=11 animals; 8n=6 animals; 9b,c=7 animals. Supplementary Figures 1a, b=14 animals; 3a=3 animals; 7a-d=1 animal; 8a,b=4 animals; 9a-l=5 animals; 10a=5 animals; 14a,c=6 animals; 14b,d=3 animals; 15a=6 animals; 15b=3 animals; 16a=6 animals; 16b=3 animals; 18b = 13 animals."
Randomization	Randomization was not possible in our study design due to the difficulty in generating genetic mouse lines and the low numbers of available experimental animals.
Blinding	Blinding was not possible in our study due to the need to confirm genotype to aid in experimental feasibility of electrophysiological experiments and the requirement for the genotype to be unmasked for the analysis of our datasets. For example many of the data can not be analyzed with masking as we count fluorescent cell bodies or axons where fluorescence is driven by Cre and the presence of fluorescence

reveals the genotype. We could not blind the application of experimental drugs in pharmacological bath applications due to the extremely low hit-rate in our experiments (for example photoresponses in the SCN were obtained from 11 total cells from over 100 recorded cells).

Reporting for specific materials, systems and methods

We require information from authors about some types of materials, experimental systems and methods used in many studies. Here, indicate whether each material, system or method listed is relevant to your study. If you are not sure if a list item applies to your research, read the appropriate section before selecting a response.

Materials & experimental systems

n/a	Involved in the study
	☒ Antibodies
☒	Eukaryotic cell lines
☒	Palaeontology and archaeology
	☒ Animals and other organisms
☒	Clinical data
☒	Dual use research of concern

Methods

n/a	Involved in the study
☒	ChIP-seq
☒	Flow cytometry
☒	MRI-based neuroimaging

Antibodies

Antibodies used

Rabbit anti-melanopsin (ATSBio - Cat#: AB-N39) Chicken anti-mCherry antibody (Abeam - Cat#: ab205402) Goat anti-GFP antibody (Abeam - CAT#: ab5450) Chicken anti-GFP antibody (Aves Labs GFP-1010) Guineapig anti-RBPMS (Phosphosolutions 1832-RBPMS) Goat anti-cholera toxin subunit B (List Labs - Cat#: 703) Rabbit anti-vasopressin antibody (lmmunostar - Cat#: 20069) Rabbit anti-opsin antibody blue (Millipore - Cat#: ab5407) Mouse anti-Neurofilament H (SMI-32 Biolegend; previously Covance Cat SMI32) Guinea Pig anti-RBPMS (Phosphosolutions Cat# 1832-RBPMS) Donkey anti-rabbit Alexa Flour 647 (Jackson lmmuno Research - Cat#: 711-545-152) Donkey anti-goat Alexa Flour 488 (Jackson lmmuno Research - Cat#: 705-545-147) Donkey anti-chicken Cy3 (Jackson lmmuno Research - Cat#: 703-165-155) Donkey anti-chicken Alexa Fluor 488 (Jackson lmmuno Research - Cat#: 7703-545-155) Donkey anti-mouse Alexa Fluor 488 (Jackson lmmuno Research - Cat#: 715-545-151) Donkey anti-mouse Alexa Fluor 594 (Jackson lmmuno Research - Cat#: 715-585-151) Donkey anti-mouse Alexa Fluor 647 (Jackson lmmuno Research - Cat#: 715-605-151) Donkey anti-Guinea Pig Alexa Fluor 488 (Jackson lmmuno Research - Cat#: 706-545-148) Donkey anti-Guinea Pig Alexa Fluor 647 (Jackson lmmuno Research - Cat#: 715-605-148).

Validation

Rabbit anti-melanopsin (Advanced targeting systemsl cat AB-n39) is an affinity-purified version of the n-38 antibody (RRID: AB_1608077) and has been previously published in 11 scientific articles first by Dumitrescu et. al. 2009 who state that it "is raised against a synthetic peptide consisting of the 15 N-terminal amino acids of mouse melanopsin (MDSPSGPRVLSSLTQ). It produces no staining in melanopsin knockout mice (Opn4 −/−)." Using the OPN4Cre mouse line we also validated this antibody produces no staining in OPN4Cre+/+ mice, which are OPN4-/- or knockout mice (Chen et. al. 2011). Chicken anti-mCherry antibody (Abcam ab205402; RRID AB2722769) is raised against the recombinant protein (His-tag) corresponding to mCherry (sequence from Shaner NC et al. Nature Biotechnology 22:1567-1572 (2004). This antibody has been published in 37 articles and stains a band with the predicted molecular weight of 30kDa in a lysate of HEK293 cells transfected with pFin-EF1-mCherry vector. Goat anti-GFP antibody (ab5450; RRID AB_304897) is a polyclonal antibody raised against the recombinant full length protein corresponding to GFP. This antibody produces signal amplification in cells expressing GFP and has been references in 10 publications. Chicken anti-GFP (Aves labs GFP-1020; RRID AB_2307313) is a polyclonal antibody raised against recombinant GFP expressed in Escherichia coli. Antibodies were analyzed by western blot analysis (1:5000 dilution) and immunohistochemistry (1:500 dilution) using transgenic mice expressing the GFP gene product. Western blots were performed using BlokHen® (Aves Labs) as the blocking reagent, and HRP-labeled goat anti-chicken antibodies (Aves Labs, Cat. #H-1004) as the detection reagent. The vendor lists this antibody as being referenced in 643 research articles. In our experiments, this antibody strongly amplifies the signal that is produced in mice where Cre drives the production of EGFP in specific neuronal populations (GlyT2Cre;Ai140; Ai140 from Jackson mouse line 030220). Goat anti-cholera toxin subunit B (List Labs - Cat#: 703; RRID AB_10013220) is a goat polyclonal antibody against the Cholera Toxin B subunit and has been cited in 137 research articles. In our experiments, this antibody produces strong amplification of the axon-terminals of retinal ganglion cells in the suprachiasmatic nucleus following eye injections of Cholera Toxin B subunit, as previously published in other papers using the same methods (Hattar et al. 2006 PMID: 16736474) and when conjugated to a secondary antibody with a far-red (Alexa 647) fluorophore. Rabbit anti-vasopressin antibody (lmmunostar - Cat#: 20069; RRID AB_572219) is a rabbit polyclonal antibody against argenine vasopressin and has 67 linked citations. The antibody produces significant fluorescent staining and significant biotin-avidin/HRP staining at a 1/2,000 – 1/4,000 dilution in rat hypothalamus. Staining is completely eliminated by pretreatment of the diluted antibody with 10 μg/mL of arginine vasopressin. Preadsorption with as much as 100 ug/mL of oxytocin had no effect on immunolabeling, confirming specificity. In our experiments this antibody labeled neurons in the suprachiasmatic nucleus, the paraventricular nucleus, and the supraoptic nucleus as published in previous studies (PMID: 34561434; PMID: 21525287; PMID: 30283813). Rabbit anti-opsin antibody blue (Millipore Sigma - Cat#: ab5407; RRID AB_177457) is a rabbit polyclonal antibody against recombinant human blue opsin. Validation information can be found on the manufacturers website: https://www.emdmillipore.com/US/en/product/Anti-Opsin-Antibody-blue,MM_NF-AB5407. In our experiments in mice this antibody stained S-cone photoreceptor outer segments located predominantly in the ventral retina as previously published (PMID: 15930394; PMID: 18076080). Mouse anti-Neurofilament H (SMI-32 Biolegend; previously Covance Cat SMI32;RRID AB_509997) is a mouse affinity purified monoclonal antibody against neurofilament H and has been referenced in 146 publications. Validation information can be found at the manufacturer's website: https://www.biolegend.com/en-us/products/purified-anti-neurofilament-h-nf-h-nonphosphorylated-antibody-11475. In our experiments SMI-32 labeled alpha-type retinal ganglion cells as previously published (PMID: 30017393; PMID: 24440397). Guinea Pig anti RBPMS (Phosphosolutions Cat# 1832-RBPMS lot NB322g) is a polyclonal antibody raised against a synthetic peptide corresponding to amino acid residues from the N-

	terminal region of rat RBPMS, conjugated to keyhole limpet hemocyanin (KLH). This antibody is validated by Pérez de Sevilla Müller and colleagues (PMID: 25631988) and in our experiments labels retinal ganglion cells but not retinal amacrine cells.

Animals and other research organisms

Policy information about studies involving animals; ARRIVE guidelines recommended for reporting animal research, and Sex and Gender in Research

Laboratory animals	Adult mice (1-6 months of age were used). Strains used were GlyT2Cre (Tg(Slc6a5-cre)KF109Gsat/Mmucd) - sperm purchased from the Mutant Mouse Resource and Research Center (Stock 030730-UCD); PACAP-2A-IRES-Cre B6.Cg-Adcyap1tm1.1(cre)Hze/ZakJ; strain 030155; The Jackson Laboratories; Ai32 (RCL-ChR2(H134R)/EYFP) - The Jackson Laboratories; Ai9 (RCL-tdT) - The Jackson Laboratories; Ai140 (TITL-GCF-ICL-tTA2) - The Jackson Laboratories; OPN4Cre mouse (tm1.1(cre)Saha/J) - S. Hattar, The Johns Hopkins University.
Wild animals	No wild animals were used in this study.
Reporting on sex	Animals of both sexes were used in this study and data from both sexes were combined. Due to the low number of animals, we did not perform sex-specific analysis. Where sex was recorded, the sex of the mouse is listed in the source data file. The overall number of mice where sex was recorded in this study was 25 Females and 29 males.
Field-collected samples	No field-collected samples were used in this study.
Ethics oversight	Experiments involving animals were in accordance with the National Institutes of Health guidelines, and all procedures were approved by the Oregon Health and Science University Institutional Animal Care and Use Committee. Protocol number TR02IP00000096.

Note that full information on the approval of the study protocol must also be provided in the manuscript.
